# Human-Computer Interaction Among Older Adults in a Mixed-Reality Technology-Based Exercise Program

**DOI:** 10.1093/geroni/igaf122.1145

**Published:** 2025-12-31

**Authors:** Michael Dino, Ladda Thiamwong, Rui Xie

**Affiliations:** University of Central Florida, University of Central Florida, Florida, United States; University of Central Florida, Orlando, Florida, United States; University of Central Florida, Orlando, Florida, United States

## Abstract

Maintaining mobility becomes increasingly critical for community-dwelling older adults’ overall health and independence. Recognizing this challenge, scholars and healthcare providers are gradually adopting technological innovations, such as mixed reality (MR), to support older adults’ physical activity and wellness. However, emerging technologies are prone to disregard due to a lack of studies supporting their adoption in practice. More importantly, studies that examine human-computer interaction between mixed reality devices for health and older adult users remain scarce. To fill this gap, a multidisciplinary team created the Mixed Reality-Oriented Virtual Coach Experience (MOVE) Project. The program highlights the utilization of a developed “virtual coach” projected via head-mounted devices. Using principles of human-centered design in technology development, the research team examined how older adults interact with the virtual agent for technology refinement and pilot. A cognitive walkthrough design was employed to capture interaction data (e.g., gaze, fixation) from a pilot cohort of six consented community older adults. The participants were instructed to wear Tobii Pro Glasses 3™ while interacting with the virtual coach projected on a large screen. Heatmaps were generated and analyzed to reveal areas of interest (AOIs). Results revealed various user fixation patterns in reference to the virtual coach’s movement and posture during physical exercise sessions and some critical insights on human-computer interaction in MR use for physical exercise programs among older adults. The outcomes of the study will be used to refine programming, movement, and physical appearance of the virtual coach to maximize interaction efficiency between the system and the user.

